# Safety and pharmacokinetics of GB1211, an oral galectin-3 inhibitor: a single- and multiple-dose first-in-human study in healthy participants

**DOI:** 10.1007/s00280-023-04513-y

**Published:** 2023-03-13

**Authors:** Vassilios Aslanis, Robert J. Slack, Alison C. MacKinnon, Catherine McClinton, Susan Tantawi, Lise Gravelle, Ulf J. Nilsson, Hakon Leffler, Ashley Brooks, Sanjeev K. Khindri, Richard P. Marshall, Anders Pedersen, Hans Schambye, Fredrik Zetterberg

**Affiliations:** 1 Galecto Biotech AB, 2200 Copenhagen N, Denmark; 2 Department of Chemistry, Lund University, 22100 Lund, Sweden; 3 Department of Laboratory Medicine, Lund University, 22100 Lund, Sweden; 4 Covance Clinical Research Unit Ltd., Leeds, UK

**Keywords:** Pharmacokinetics, Drug development–phase 1, Drug development–randomized controlled trial, Clinical pharmacology–drug safety

## Abstract

**Purpose:**

Galectin-3, a β-galactoside-binding lectin, plays a key role in several cellular pathways involved in chronic inflammation, heart disease and cancer. GB1211 is an orally bioavailable galectin-3 inhibitor, developed to be systemically active. We report safety and pharmacokinetics (PK) of GB1211 in healthy participants.

**Methods:**

This phase 1, double-blind, placebo-controlled, first-in-human study (NCT03809052) included a single ascending-dose phase (with a food-effect cohort) where participants across seven sequential cohorts were randomized 3:1 to receive oral GB1211 (5, 20, 50, 100, 200 or 400 mg) or placebo. In the multiple ascending-dose phase, participants received 50 or 100 mg GB1211 or placebo twice daily for 10 days. All doses were administered in the fasted state except in the food-effect cohort where doses were given 30 min after a high-fat meal.

**Results:**

All 78 participants received at least one GB1211 dose (*n* = 58) or placebo (*n* = 20) and completed the study. No safety concerns were identified. Following single and multiple oral doses under fasted conditions, maximum GB1211 plasma concentrations were reached at 1.75–4 h (median) post-dose; mean half-life was 11–16 h. There was a ~ twofold GB1211 accumulation in plasma with multiple dosing, with steady-state reached within 3 days; 30% of the administered dose was excreted in urine as unchanged drug. Absorption in the fed state was delayed by 2 h but systemic exposure was unaffected.

**Conclusion:**

GB1211 was well tolerated, rapidly absorbed, and displayed favorable PK, indicating a potential to treat multiple disease types. These findings support further clinical development of GB1211.

**Clinical trial registration:**

The study was registered with ClinicalTrials.gov (identifier: NCT03809052).

**Supplementary Information:**

The online version contains supplementary material available at 10.1007/s00280-023-04513-y.

## Introduction

Galectin-3 is a β-galactoside-binding lectin, which plays an important role in cell proliferation, differentiation, angiogenesis and apoptosis, and is a key regulator of fibrosis and inflammation in the lungs, liver and kidneys [1–3]. As well as having a prominent role in chronic inflammation, galectin-3 has been implicated in the development of several cancers, including high fatality cancers such as non-small cell lung cancer (NSCLC) [4, 5], where galectin-3 has been shown to down-regulate T cell function and promote M2 tumor-associated macrophage (TAM) activation [6–8]. In addition, galectin-3 is involved in many other disease conditions including heart disease, kidney disease, viral infections, and autoimmune and neurodegenerative disorders [9–13]. Previous studies have also indicated galectin-3 as a diagnostic or prognostic biomarker in monitoring disease progression or response to therapy in certain types of heart disease, kidney disease and cancer [14–17]. The circulating galectin-3 concentration in the serum of cancer patients has been reported to be higher than that of healthy individuals, and the level of galectin-3, which can vary across different human tumors, has been shown to correlate with tumor progression [18, 19].

There is interest surrounding the therapeutic potential for galectin-3 inhibition in fibrotic disease [5]. Galectin-3 is highly expressed in fibrotic tissue of diverse etiologies, regulates myofibroblast differentiation (the major source of collagens during tissue fibrosis) and has profound effects on macrophage polarization. Macrophage secretion of galectin-3 results in the activation of myofibroblasts, further amplifying a sustained pro-fibrotic loop [2, 20]. Diminished fibrotic severity and decreased scarring following injury have been observed in galectin-3-deficient mice, with the absence of galectin-3 resulting in reduced myofibroblast activation and accumulation in the lungs and kidneys [21, 22]. Similarly, galectin-3 inhibitors, when delivered locally in the murine lung, can markedly reduce fibrosis [22, 23]. Upregulation of galectin-3 has been observed in fibroproliferative areas of the lung in patients with interstitial pneumonia, with serum levels rising significantly during an acute exacerbation [22].

Galectin-3 inhibition as treatment for fibrotic illness has recently been observed in human studies. GB0139 (also known as TD139), is a potent thiodigalactoside inhibitor of galectin-3 [24], and a phase 1/2a trial (ClinicalTrials.gov identifier: NCT02257177) evaluated the pharmacokinetic (PK) and pharmacodynamic (PD) profile of GB0139 in healthy individuals and patients with idiopathic pulmonary fibrosis (IPF). Inhaled GB0139 suppressed galectin-3 expression and decreased plasma biomarkers associated with IPF disease progression [25]. Also, preliminary analyses of inhaled GB0139 indicated trends towards meaningful reductions in fibrotic disease-associated biomarkers in hospitalized patients with coronavirus disease 2019 [26]. While these studies demonstrate the potential for GB0139 as a treatment for fibrotic disease, the inhaled formulation offers a lung-specific therapeutic effect. Systemic inhibition of galectin-3 is required to treat fibrosis across other organs and a wider variety of conditions.

The small molecule galectin-3 inhibitor, GB1211, has been developed amongst a new class of galactopyranosides, whereby non-natural aromatic substituents are introduced to the 1- and 3-position of α-D-galactopyranosides, and affinity subsequently optimized by specific interactions, such as fluorine-amide, phenyl-arginine, sulphur-π, and halogen bonds [27]. GB1211 has high affinity for galectin-3 (0.025 ± 0.0017 µM) with good oral bioavailability in animals (68% in mice) [27] and is currently in development for the treatment of cancer and fibrotic disorders including NSCLC (identifier: NCT05240131) and cirrhosis (identifier: NCT05009680), respectively [5]. Support for this comes from galectin-3 inhibition with the GB1211 structural analogue GB1107 which leads to a reduction in M2-like TAMs and increased infiltration and activity of CD8 + cytotoxic T lymphocytes within NSCLC tumors [7]. This results in reduced tumor growth and metastasis and increased response to anti-programmed death ligand 1 (PD-L1) immunotherapy [7]. In addition, GB1211 reverses the galectin-3-induced inhibition of immune checkpoint inhibitors (pembrolizumab and atezolizumab) binding to programmed cell death protein 1 and PD-L1, thereby reducing tumor resistance to these agents and restoring response to immune checkpoint therapy [28]. As a result of these findings, the safety and efficacy of GB1211 is being investigated in combination with atezolizumab in NSCLC (identifier: NCT05240131).

We report the results of a phase 1 trial of GB1211; the first clinical study of GB1211. The aim of this study was to evaluate the safety, tolerability and PK of oral GB1211 in healthy participants.

## Materials and methods

### Primary and secondary objectives

This was a randomized, double-blind, placebo-controlled, first-in-human study in healthy adults. The primary objective was to assess the safety and tolerability of single and multiple doses of GB1211. The secondary objectives were to evaluate the PK of single and multiple doses of GB1211 in participants’ plasma and urine (single dose PK only were evaluated in the urine), and to determine the effect of food intake on the single dose PK.

### Conduct and ethics

The study took place from January–June 2019 at the Covance Clinical Research Unit (Leeds, UK) and was conducted in accordance with the principles of the Declaration of Helsinki and the International Conference on Harmonisation and Good Clinical Practice. An independent ethics committee reviewed and approved the study protocol and its amendments. All participants freely gave their written informed consent before starting the study. The study was registered with ClinicalTrials.gov (identifier: NCT03809052).

### Study design

The study comprised two parts: Part A was a single ascending-dose phase, incorporating a single-cohort, randomized, 2-part arm to investigate the effect of food; Part B was a multiple ascending-dose phase (Supplementary Fig. S1).

#### Part A: randomized, double-blind, placebo-controlled, single-ascending dose phase

A starting dose of 5 mg was chosen as this was approximately twofold lower than the maximum recommended starting dose (MRSD) in humans of 9.6 mg determined from the results of a murine PD study, and was therefore not expected to have any pharmacological activity. This dose was also more than 30-fold lower than the MRSD of 162 mg calculated from the no observed adverse effect level in the toxicology species (mouse and dog). It was therefore considered to be a well tolerated and pharmacologically inactive starting dose allowing a wide dose escalation range.

Participants in Part A were sequentially assigned to 1 of 5 cohorts (cohorts A1–A5; 5 mg, 20 mg, 50 mg, 100 mg or 200 mg; *n* = 8 in each group) and randomized 3:1 within each cohort to receive GB1211 (*n* = 6) as 5 mg or 50 mg capsule formulations or matched placebo (*n* = 2). A further two cohorts were recruited. In cohort A6, 8 participants received either 50 mg GB1211 (10 × 5 mg capsules) or placebo without sentinel dosing to assess the effect of capsule number/size on systemic exposure, through comparison with exposure in fasted cohort A3 participants receiving 50 mg as 1 × 50 mg capsule. In cohort A7, 8 participants received 400 mg GB1211 (8 × 50 mg capsules) or placebo as systemic exposure was predicted to not exceed the dose-stopping criteria. Each individual participated in one treatment period only, with the exception of the food-effect cohort (cohort A3) in which there were two treatment periods separated by at least 7 days washout. Participants resided at the Clinical Research Unit (CRU) from 1 day ahead of dosing (day − 1) to day 3 and returned for a follow-up visit 5–7 days after their final dose. For all cohorts, doses were administered in the fasted state in the morning of day 1 of Treatment Period 1. In the food-effect cohort, doses were given 30 min after the start of a high-fat breakfast (973 calories total: approximately 150 protein calories, 250 carbohydrate calories, and 500–600 fat calories) on day 1 of Treatment Period 2. For cohorts A1 to A5 in Treatment Period 1, dosing occurred such that one pair (one active and one placebo) were dosed at least 24 h before the remaining participants. Continuation to dose those who remained was at the investigator’s discretion. Escalating doses were administered following satisfactory sponsor and investigator review of the safety and tolerability data (up to 48 h post-final dose) and PK data (up to 24 h post-final dose) from the lower dose levels.

#### Part B: randomized, double-blind, placebo-controlled, multiple-ascending dose phase

Cohort B comprised 22 participants in two cohorts, B1 and B2, with 8 and 3 participants in each cohort randomized to receive GB1211 or placebo, respectively. Following review of safety, tolerability, and PK data from cohorts A1–A6, participants received twice-daily doses (BID) under fasted conditions on days 1–9 and a final single dose in the morning of day 10. Participants in B1 received 50 mg GB1211 (1 × 50 mg capsule) BID or placebo and B2 received 100 mg GB1211 (2 × 50 mg capsules) BID or placebo. There was one treatment period and all participants stayed at the CRU from day − 1 until the morning of day 11 (24 h after the final dose on day 10). All were to return for a follow-up visit 5–7 days after their final dose.

### Participants

Screening for study eligibility occurred within 28 days prior to the first dose administration. Healthy males or females of any ethnic origin, aged 18–55 years for Part A and 18–60 years for Part B, weighing ≥ 50 kg with a body mass index (BMI) of 18.0–32.0 kg/m^2^ inclusive, were eligible for participation. Key exclusion criteria included a history or presence of any disease or clinical manifestation that might influence study participation or results; significant hypersensitivity or intolerance to any substance; or any condition known to interfere with the absorption and/or excretion of orally administered drugs. Participants were also excluded if they had clinically significant abnormalities in electrocardiogram (ECG) findings or used products deemed inappropriate for recent/concomitant use during the study. Full exclusion criteria can be found in the Supplementary materials.

### Safety and tolerability outcomes

Safety and tolerability assessments included adverse event (AE) monitoring, physical examinations, laboratory assessments (hematology, clinical chemistry, and urinalysis), vital signs (blood pressure, pulse rate, and body temperature) and ECG.

### Blood and urine collection for PK assessments

Blood samples (4 mL) were collected in K3 EDTA tubes by venipuncture or cannulation. In Part A, samples were taken pre-dose and at 0.5, 1, 1.5, 2, 3, 4, 6, 8, 12, 16, 24, 36 and 48 h post-dose. In part B, samples were taken pre-dose on days 1 (pre-AM dose) and 10 (pre-final dose) and at 0.5, 1, 1.5, 2, 3, 4, 6, 8 and 12 h post-AM dose for day 1 and post-final dose for day 10. Samples were also taken on days 4–9 pre-AM dose, on day 11 at 24 and 36 h post-final dose, and on day 12 at 48 h post-final dose. Blood samples were centrifuged at 1500 g for 10 min at − 20 °C and split into two plasma aliquots.

Urine was collected by spot collection in Part A only before dosing and between 0–6, 6–12, 12–24, and 24–48 h post-dose. Blood and urine aliquots were stored in polypropylene tubes at − 70 °C until bioanalysis. Plasma and urine concentrations of GB1211 were measured using validated liquid chromatography methods with tandem mass spectrometry detection with lower limits of quantification of 0.500 ng/mL in both biological media.

### PK analyses

Noncompartmental PK analysis was performed on individual plasma and urine concentration data, using Phoenix^®^ WinNonlin^®^ 8.1 software (Certara USA Inc., Princeton NJ). Plasma PK parameter endpoints for Part A included area under the plasma concentration–time curve (AUC) from time 0 to infinity (AUC_0–∞_), AUC from time 0 to the time of the last quantifiable concentration (AUC_0–tlast_), maximum observed plasma concentration (C_max_), time of the maximum observed plasma concentration (T_max_), terminal half-life in plasma (t_½_), apparent total plasma clearance (CL/F), and apparent volume of distribution (V_z_/F). Urine PK parameters calculated in Part A included amount of drug excreted in urine (A_e_), percentage of dose excreted unchanged in urine (f_e_) and renal clearance (CL_R_).

In Part B, the plasma PK parameters included AUC over a dosing interval (AUC_0–τ_), AUC_0–∞_, C_max_, T_max_, t_½_, minimum observed plasma concentration (C_min_), observed accumulation ratio based on AUC_0–τ_ (RA_AUC0–τ_) and observed accumulation ratio based on C_max_ (RA_Cmax_). Further details can be found in the Supplementary materials.

### Statistical methods

The safety analysis population included all participants who received at least 1 dose of either GB1211 or placebo. No formal statistical assessment was performed for the safety analysis.

The PK population comprised all participants who received at least 1 GB1211 dose and had at least one quantifiable GB1211 concentration. Dose proportionality was analyzed for exposure parameters after fasted doses. The power model was used on AUC_0–∞_, AUC_0–tlast_, and C_max_ on day 1 in Part A. Data were analyzed over two dose ranges: 50–400 mg (50 mg capsules) and 5–50 mg (5 mg capsules). An analysis of variance was used on AUC_0–τ_ and C_max_ on day 10 in Part B. The effect of food on AUC_0–∞_, AUC_0–tlast_, and C_max_ at 50 mg in Part A was investigated using a mixed model which included treatment as a fixed effect and subject as a random effect.

## Results

### Participant disposition and baseline characteristics

A total of 78 participants aged 19–60 years enrolled in the study. Mean (standard deviation [SD]) BMI was 25.5 (3.41) kg/m^2^ for cohorts in Part A and 25.2 (3.40) kg/m^2^ for those in Part B. The majority of participants (65.4%) were male. Full baseline demographics and characteristics are available in Table 1. All participants who enrolled in the trial received at least 1 dose of the study drug (*n* = 58) or placebo (*n* = 20) and completed the study.

**Table 1:** Baseline demographics and characteristics for Part A (a) and Part B (b) – safety population

(a)								
	Placebo (*n* = 14)	GB1211						
		5 mg (1 × 5 mg), fasted (*n* = 6)	20 mg (4 × 5 mg), fasted (*n* = 6)	50 mg (10 × 5 mg), fasted (*n* = 6)	50 mg (1 × 50 mg), fasted/fed (*n* = 6)	100 mg (2 × 50 mg), fasted (*n* = 6)	200 mg (4 × 50 mg), fasted (*n* = 6)	400 mg (8 × 50 mg), fasted (*n* = 6)
Age, years; median (range)	35 (19–55)	34 (23–42)	39 (24–52)	27 (23–54)	25 (20–39)	35 (21–45)	35 (21–53)	42 (22–49)
Sex, male; *n* (%)	8 (57.1)	3 (50.0)	4 (66.7)	4 (66.7)	4 (66.7)	5 (83.3)	5 (83.3)	4 (66.7)
Race; *n* (%)								
Asian	–	1 (16.7)	1 (16.7)	–	–	1 (16.7)	–	–
Black	3 (21.4)	–	1 (16.7)	1 (16.7)	–	–	–	–
White	11 (78.6)	4 (66.7)	4 (66.7)	5 (83.3)	5 (83.3)	5 (83.3)	6 (100)	6 (100)
Multiple	–	1 (16.7)	–	–	1 (16.7)	–	–	–
Ethnicity; *n* (%)								
Hispanic or Latino	–	–	–	–	–	–	–	–
Not Hispanic or Latino	14 (100)	6 (100)	6 (100)	6 (100)	6 (100)	6 (100)	6 (100)	6 (100)
Weight, kg; mean (SD)	81.3 (12.82)	75.9 (7.27)	77.9 (19.03)	73.3 (16.72)	71.4 (7.87)	75.1 (13.94)	73.7 (11.61)	79.7 (12.63)
Height, cm; mean (SD)	173.6 (10.26)	172.3 (10.31)	171.7 (9.65)	176.2 (12.86)	169.8 (5.00)	175.3 (5.20)	174.7 (8.45)	173.7 (8.12)
BMI, kg/m^2^; mean (SD)	26.9 (3.06)	25.9 (4.48)	26.1 (3.91)	23.3 (2.86)	24.8 (2.73)	24.5 (4.63)	24.1 (3.25)	26.2 (1.90)

(b)			
	Placebo BID (*n* = 6)	GB1211	
		50 mg BID (*n* = 8)	100 mg BID (*n* = 8)
Age, years; median (range)	40 (27–50)	46 (22–54)	34 (25–60)
Sex, male; *n* (%)	4 (66.7)	5 (62.5)	5 (62.5)
Race; *n* (%)			
White	6 (100)	8 (100)	8 (100)
Ethnicity; *n* (%)			
Hispanic or Latino	–	–	–
Not Hispanic or Latino	6 (100)	8 (100)	8 (100)
Weight, kg; mean (SD)	70.9 (13.24)	85.4 (17.20)	76.8 (9.52)
Height, cm; mean (SD)	175.2 (5.71)	178.9 (7.62)	173.4 (4.21)
BMI, kg/m^2^; mean (SD)	23.0 (3.50)	26.5 (3.78)	25.5 (2.35)

*BID* twice daily, *BMI* body mass index, *SD* standard deviation

### Safety and tolerability

There were no serious AEs or severe AEs reported, and no study discontinuations due to AEs reported in the treatment-emergent period (the period of time from the first dose of study drug to the date of last dose + 30 days). Treatment-emergent AEs (TEAEs) are summarized in Table 2.

**Table 2:** Summary of treatment-emergent adverse events for Part A (a) and Part B (b) – safety population

(a)										
*n* (%)	Placebo		GB1211							
	Fasted (*n* = 14)	Fed (*n* = 2)	5 mg (1 × 5 mg), fasted (*n* = 6)	20 mg (4 × 5 mg), fasted (*n* = 6)	50 mg (10 × 5 mg), fasted (*n* = 6)	50 mg (1 × 50 mg), fasted (*n* = 6)	50 mg (1 × 50 mg), fed (*n* = 6)	100 mg (2 × 50 mg), fasted (*n* = 6)	200 mg (4 × 50 mg), fasted (*n* = 6)	400 mg (8 × 50 mg), fasted (*n* = 6)
Participants with TEAEs	5 (35.7)	1 (50.0)	3 (50.0)	1 (16.7)	1 (16.7)	1 (16.7)	1 (16.7)	1 (16.7)	0	1 (16.7)
Participants with SAEs	0	0	0	0	0	0	0	0	0	0
Participants discontinued due to TEAEs	0	0	0	0	0	0	0	0	0	0
Number of TEAEs	5	1	4	1	1	1	1	2	0	1
Severity (all TEAEs)										
Mild	5 (35.7)	1 (50.0)	3 (50.0)	1 (16.7)	1 (16.7)	1 (16.7)	0	1 (16.7)	0	1 (16.7)
Moderate	0	0	0	0	0	0	1 (16.7)	0	0	0
Severe	0	0	0	0	0	0	0	0	0	0
Total	5 (35.7)	1 (50.0)	3 (50.0)	1 (16.7)	1 (16.7)	1 (16.7)	1 (16.7)	1 (16.7)	0	1 (16.7)
Severity (possibly related to study treatment)										
Mild	3 (21.4)	0	1 (16.7)	0	0	0	0	1 (16.7)	0	1 (16.7)
Moderate	0	0	0	0	0	0	0	0	0	0
Severe	0	0	0	0	0	0	0	0	0	0
Total	3 (21.4)	0	1 (16.7)	0	0	0	0	1 (16.7)	0	1 (16.7)

(b)			
*n* (%)	Placebo BID (*n* = 6)	GB1211	
		50 mg BID (*n* = 8)	100 mg BID (*n* = 8)
Participants with TEAEs	1 (16.7)	4 (50.0)	3 (37.5)
Participants with SAEs	0	0	0
Participants discontinued due to TEAEs	0	0	0
Number of TEAEs	2	8	8
Severity (all TEAEs)			
Mild	1 (16.7)	2 (25.0)	3 (37.5)
Moderate	0	2 (25.0)	0
Severe	0	0	0
Total	1 (16.7)	4 (50.0)	3 (37.5)
Severity (possibly related to study treatment)			
Mild	1 (16.7)	2 (25.0)	3 (37.5)
Moderate	0	2 (25.0)	0
Severe	0	0	0
Total	1 (16.7)	4 (50.0)	3 (37.5)

*BID* twice daily, *SAE* serious adverse event, *TEAE* treatment-emergent adverse event

For adverse events that change severity rating, the adverse event will be included only once under the maximum severity rating

In Part A, 14 (25.0%) participants experienced 17 TEAEs: 6 participants with 6 TEAEs in the placebo group and 8 participants with 11 TEAEs in the GB1211 group. There was no apparent treatment- or dose-related trend and no effect of food intake or capsule formulation on TEAEs was observed. All TEAEs were classified as mild, with the exception of 1 moderate event reported in 1 participant who received 50 mg GB1211 in the fed state. In Part B, 18 TEAEs were reported amongst 8 (36.4%) participants: 2 TEAEs reported in 1 participant on placebo, and 16 TEAEs reported in 7 patients on GB1211. Two TEAEs were considered moderate, both occurring in the 50 mg BID GB1211 cohort. All other TEAEs were considered mild.

The most frequent TEAEs by system organ class (SOC) in Part A were gastrointestinal disorders (4 individuals reporting four TEAEs: constipation, diarrhea, dry mouth, dyspepsia), followed by skin and subcutaneous tissue disorders (3 individuals reporting three TEAEs: dry skin, rash). All other TEAEs by SOC were experienced by ≤ 2 participants (Supplementary Table S1a). In Part B, the most frequent TEAEs by SOC were also gastrointestinal disorders (3 participants reporting six TEAEs: constipation, abdominal distension, abdominal pain, abdominal pain upper), followed by nervous system disorders (4 participants reporting five TEAEs: headache), and renal and urinary disorders (3 participants reporting three TEAEs: pollakiuria, dysuria). All other TEAEs by SOC in Part B were experienced by one individual only (Supplementary Table S1b).

There was no treatment- or dose-related trend identified in clinical laboratory evaluation, vital signs, ECG or physical examination following single or multiple GB1211 doses.

Six participants (GB1211, *n* = 3; placebo, *n* = 3) in Part A and 8 participants (GB1211, *n* = 7; placebo, *n* = 1) in Part B experienced a TEAE considered by the principal investigator to be possibly related to treatment. The related TEAEs reported following GB1211 in Part A were diarrhea, dyspepsia and rash, and in Part B were headache, constipation, pollakiuria, abdominal distension, abdominal pain upper, dysuria and menstruation irregularity.

### PK analysis

#### Single oral dose plasma PK of GB1211 in the fasted state

**Fig. 1:**
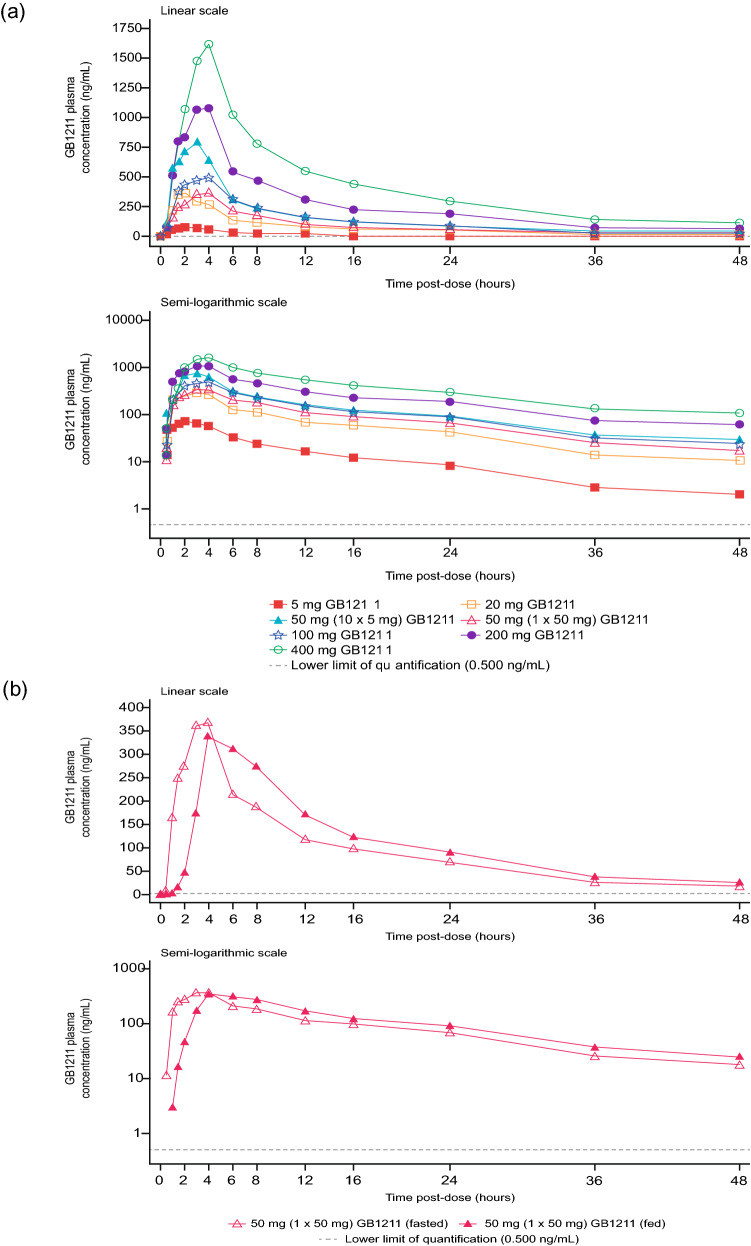
Mean plasma concentrations of GB1211 following **A** single oral fasted doses (5–400 mg) and **B** single oral doses (50 mg) in the fasted and fed state

GB1211 was rapidly absorbed with median T_max_ ranging from 1.75 to 2.00 h post-dose across the 5–50 mg (5 mg capsule) dose range (Table 3a). The absorption peak was delayed for the 50 mg capsule, with median T_max_ ranging from 3.04 to 4.00 h across the 50–400 mg (50 mg capsule) dose range in fasted patients. After reaching C_max_, GB1211 plasma concentrations appeared to decline in a generally biphasic manner (Fig. 1a). Arithmetic mean terminal half-life (t_½_) ranged from 11.3 to 15.5 h with no clear trend across doses (Table 3a).

**Table 3:** Summary of the pharmacokinetic parameters for GB1211 following single oral doses (a) and multiple oral doses (b)

(a)								
Parameter	5 mg (1 × 5 mg), fasted (*n* = 6)	20 mg (4 × 5 mg), fasted (*n* = 6)	50 mg (10 × 5 mg), fasted (*n* = 6)	50 mg (1 × 50 mg), fasted (*n* = 6)	50 mg (1 × 50 mg), fed (*n* = 6)	100 mg (2 × 50 mg), fasted (*n* = 6)	200 mg (4 × 50 mg), fasted (*n* = 6)	400 mg (8 × 50 mg), fasted (*n* = 6)
AUC_0–∞_ (h·ng/mL)	712 (24.5)	3350 (10.2)	7480 (42.6)	4520 (43.2)	5420 (21.5)	6380 (21.1)	12,900 (33.7)	21,600 (25.0)
AUC_0–tlast_ (h·ng/mL)	677 (23.4)	3150 (10.2)	6890 (41.0)	4180 (45.0)	4930 (23.4)	5910 (18.8)	11,500 (36.5)	19,000 (29.4)
C_max_ (ng/mL)	75.2 (23.7)	365 (25.3)	861 (48.4)	358 (54.6)	389 (37.9)	525 (23.6)	1010 (54.1)	1550 (36.2)
T_max_^a^ (h)	2.00 (1.00, 3.00)	1.75 (1.50, 2.00)	1.75 (1.00, 4.00)	3.04 (2.00, 4.05)	5.00 (4.00, 6.00)	3.50 (1.50, 4.00)	3.50 (3.00, 4.00)	4.00 (3.00, 4.00)
t_½_^b^ (h)	11.3 (1.68)	12.2 (1.67)	13.8 (1.33)	12.7 (2.46)	12.9 (2.26)	12.8 (3.39)	15.5 (2.56)	15.2 (3.18)
CL/F (L/h)	7.02 (24.5)	5.97 (10.2)	6.69 (42.6)	11.1 (43.2)	9.23 (21.5)	15.7 (21.1)	15.5 (33.7)	18.5 (25.0)
V_z_/F (L)	114 (21.6)	104 (17.8)	133 (36.9)	200 (58.2)	169 (31.3)	282 (25.1)	344 (52.2)	398 (44.3)
CL_R_ (L/h)	2.12 (44.2)	1.90 (23.5)	1.94 (63.1)	2.24 (31.8)	2.20 (44.0)	2.59 (18.9)	1.83 (37.6)	2.67 (14.3)
A_e_ (0–48 h) (mg)	1.43 (27.0)	6.00 (19.7)	13.4 (38.7)	9.34 (44.1)	10.9 (33.5)	15.3 (34.8)	21.0 (28.0)	50.7 (33.6)
f_e_ (0–48 h) (%)	28.7 (27.0)	30.0 (19.7)	26.8 (38.7)	18.7 (44.1)	21.7 (33.5)	15.3 (34.8)	10.5 (28.0)	12.7 (33.6)

(b)				
Parameter	50 mg BID, fasted (*n* = 8)		100 mg BID, fasted (*n* = 8)	
	Day 1	Day 10	Day 1	Day 10
AUC_0-τ_ (h·ng/mL)	2220 (23.3)	5130 (29.0)	3560 (42.9)	7530 (21.1)
C_max_ (ng/mL)	330 (31.8)	658 (35.4)	546 (53.5)	975 (21.9)
C_min_ (ng/mL)	NA	249 (26.2)	NA	338 (27.5)
T_max_^a^ (h)	4.00 (1.50, 4.00)	2.50 (1.50, 4.00)	3.00 (2.00, 4.00)	3.00 (2.00, 4.00)
t_½_^b^ (h)	NR	21.4 (5.81)	NR	13.1 (2.65)
CL/F (L/h)	NR	9.75 (29.0)	NR	13.3 (21.1)
V_z_/F (L)	NR	311 (51.8)	NR	248 (28.2)
RA_Cmax_	NA	2.00 (43.8)	NA	1.79 (39.6)
RA_AUC0–t_	NA	2.31 (32.9)	NA	2.11 (31.4)

*A*_*e*_* (0–48 h)* amount of drug excreted unchanged in urine from 0 to 48 h postdose, *AUC*_*0–τ*_ area under the plasma concentration–time curve over a dosing interval (τ = 12 h), *AUC*_*0–tlast*_ area under the plasma concentration–time curve from time 0 to the time of last quantifiable concentration, *AUC*_*0–∞*_ area under the plasma concentration–time curve from time zero to infinity, *BID* twice daily, *CL/F* apparent total plasma clearance, *CL*_*R*_ renal clearance, *C*_*max*_ maximum observed plasma concentration, *C*_*min*_ minimum observed plasma concentration, *CV* coefficient of variation, *f*_*e*_* (0–48 h)* percentage of dose excreted unchanged in urine from 0 to 48 h postdose, *NA* not applicable, *NR* not reported, *RA*_*AUCo–τ*_ observed accumulation ratio based on AUC_0-τ_, *RA*_*Cmax*_ observed accumulation ratio based on C_max_, *SD* standard deviation, *t*_*½*_ apparent plasma terminal elimination half-life, *T*_*max*_ time of maximum observed plasma concentration, *V*_*z*_*/F* apparent volume of distribution during the terminal phase. Geometric mean (CV%) data are presented

^a^Median (min–max)

^b^Arithmetic mean (SD)

Systemic exposure to GB1211 was notably higher following administration of 50 mg as 10 × 5 mg capsules compared with a single 50 mg capsule, with geometric mean AUC_0–tlast_, AUC_0–∞_ and C_max_ being 1.6-, 1.7- and 2.4-fold higher, respectively.

Low to moderate between-subject variability was noted for AUC_0–tlast_, AUC_0–∞_ and C_max_, with geometric coefficient of variation (CV%) ranging from approximately 10.2–45.0%, 10.2–43.2% and 23.6–54.6%, respectively, across all treatments.

#### Single oral dose plasma PK of GB1211 in the fed state (food-effect assessment)

Following 50 mg GB1211 administration in the fed state, a lag time in the appearance of GB1211 in plasma was observed (Fig. 1b). Concentrations were first quantifiable at times ranging from 1.00 to 3.00 h post-dose. Maximum plasma concentrations were obtained at a median T_max_ of 5.00 h post-dose, ~ 2 h later than those observed in the fasted state. AUC_0-tlast_, and C_max_ were higher following intake of 50 mg GB1211 in the fed vs fasted state (4930 vs 4180 h·ng/mL and 389 vs 358 ng/mL, respectively; Table 3a). Statistical analysis of the effect of food on PK parameters is summarized in Supplementary Table S2.

#### Single oral dose urinary PK of GB1211

The geometric mean fraction of the dose excreted in the urine as unchanged drug over 48 h post-dose (fe_(0–48 h)_) ranged from 26.8–30.0% for the 5–50 mg dose range (5 mg capsule). Across the 50–400 mg dose range (50 mg capsules; fasted state), fe_(0–48 h)_ was lower, ranging from approximately 10.5–18.7%, indicating a possible reduced absorption with the 50 mg capsule compared with the 5 mg capsule formulation (Table 3a). Renal clearance appeared to be dose-independent, with geometric mean CL_R_ estimates ranging from 1.83 to 2.67 L/h across all doses*.* The CL_R_ was greater than glomerular filtration rate (GFR) when corrected for the fraction of unbound GB1211 (f_u_) in plasma (approximately 0.4 L/h; GFR approximately 7.5 L/h and GB1211 f_u_ of 5.34%) suggesting that GB1211 undergoes active secretion in the kidney.

#### Plasma PK following multiple oral doses

**Fig. 2:**
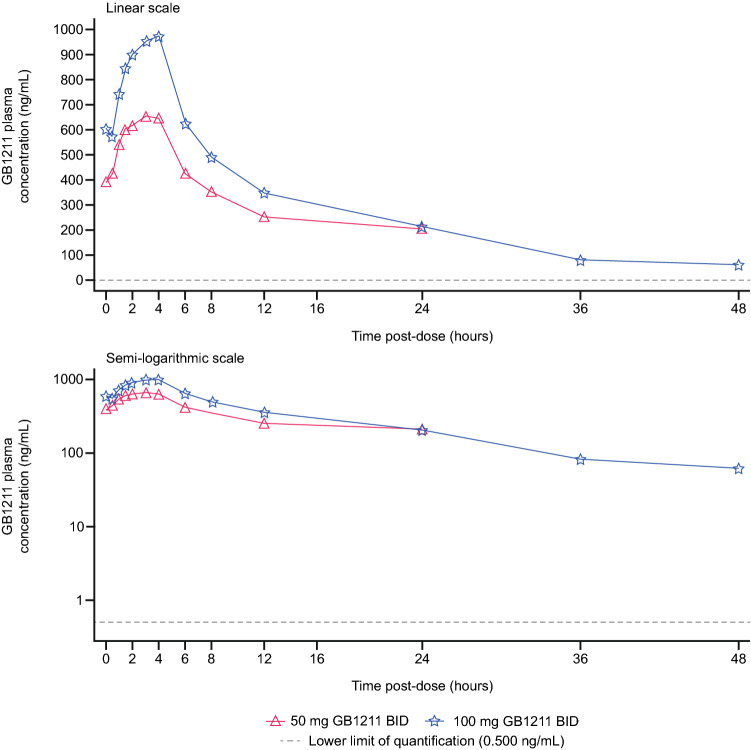
Mean plasma concentrations of GB1211 following multiple oral doses – study day 10 *BID* twice daily

After administration of multiple BID doses of GB1211, plasma concentrations peaked at a median T_max_ of 2.50–4.00 h post-dose on days 1 and 10, with no trend observed across doses or between days. On day 10, after reaching C_max_, GB1211 plasma concentrations declined in an apparently biphasic manner (Fig. 2) with mean t_½_ of approximately 21.4 and 13.1 h at 50 and 100 mg BID, respectively (Table 3b).

Steady-state GB1211 plasma concentrations were reached by the morning of day 3, 48 h after the start of the BID multiple-dosing period*.* Steady-state trough (pre-morning dose) plasma concentrations were similar across all days until the last sampling period on day 10. Corresponding geometric mean (range) trough (12 h post-dose) concentrations on day 10 (represented by C_min_) were 249 (184–381) ng/mL and 338 (203–497) ng/mL for 50 and 100 mg BID doses, respectively. Between-subject variability was generally low to moderate for AUC_0–τ_ and C_max_, with geometric CV% values ranging from approximately 21.1–42.9% and 21.9–53.5%, respectively, across days 1 and 10. Accumulation of GB1211 in plasma based on geometric mean AUC_0-τ_ (RA_AUC0–τ_) by day 10 was of 2.31 for 50 mg BID, and of 2.11 for 100 mg BID doses*.* Accumulation based on geometric mean C_max_ (RA_Cmax_) was of similar magnitude: 2.00 for 50 mg BID and 1.79 for 100 mg BID (Table 3b). The geometric mean CL/F and V_z_/F estimates on day 10 were similar to those at the same dose level after single dosing in Part A.

#### Dose-proportionality assessment

After a single administration of GB1211 at 5, 20 and 50 mg (5 mg capsule) doses, AUC_0-tlast_, AUC_0-∞_ and C_max_ appeared to increase in a dose-proportional manner. This was confirmed by statistical analysis, with slope estimates (95% confidence intervals [CI]) from the regression analysis for AUC_0-tlast_, AUC_0-∞_ and C_max_ of 1.02 (0.873–1.16), 1.03 (0.881–1.18) and 1.06 (0.894–1.24), respectively (Table 4a). Across the 50–400 mg GB1211 (50 mg capsule) dose range (fasted), systemic exposure appeared to increase in sub-proportional manner: over the eightfold dose range, there were 4.5-, 4.8- and 4.3-fold increases in geometric mean AUC_0-tlast_, AUC_0-∞_ and C_max_, respectively. The deviation from dose proportionality was confirmed by statistical analysis, with slopes estimates (95% CI) from the regression analysis for AUC_0-∞_, AUC_0-tlast_ and C_max_ of 0.778 (0.612–0.945), 0.751 (0.577–0.925) and 0.730 (0.510–0.949), respectively (Table 4a).

**Table 4:** Statistical assessment of dose proportionality of the pharmacokinetic parameters of GB1211 following single oral doses (a) and multiple oral doses (b)

(a)					
GB1211 dose range	Parameter	Slope	90% CI for the ratio (100 mg: 50 mg)		Pooled geometric CV% between-participant
			Lower	Upper	
50–400 mg (50 mg capsules); fasted	AUC_0–∞_ (h·ng/mL)	0.778	0.612	0.945	31.7
	AUC_0–tlast_ (h·ng/mL)	0.751	0.577	0.925	33.6
	C_max_ (ng/mL)	0.730	0.510	0.949	43.6
5–50 mg (5 mg capsules)	AUC_0–∞_ (h·ng/mL)	1.03	0.881	1.18	28.6
	AUC_0–tlast_ (h·ng/mL)	1.02	0.873	1.16	27.5
	C_max_ (ng/mL)	1.06	0.894	1.24	33.9

(b)								
Parameter	GB1211 dose	*n*	Geometric LS means	Ratio of geometric LS means (100 mg: 50 mg)	90% CI for the ratio (100 mg: 50 mg)		*p-*value	Between-participant geometric CV%
					Lower	Upper		
AUC_0-τ_ (norm)	50 mg BID	8	8680	0.661	0.523	0.837	0.0078	27.2
	100 mg BID	8	5740					
C_max_ (norm)	50 mg BID	8	1110	0.667	0.521	0.853	0.0118	28.6
	100 mg BID	8	743					

*AUC*_*0–τ*_ area under the plasma concentration–time curve over a dosing interval (τ = 12 h), *AUC*_*0–tlast*_ area under the plasma concentration–time curve from time 0 to the time of last quantifiable concentration *AUC*_*0–∞*_ area under the plasma concentration–time curve from time zero to infinity, *BID* twice daily, *C*_*max*_ maximum observed plasma concentration, *CV* coefficient of variation, *LS* least-squares, *(norm)* normalized for dose and weight (mg/kg)

The ratio and corresponding confidence limits are back-transformed from the difference and confidence limits calculated on the log scale

After repeated GB1211 administrations, geometric mean AUC_0–τ_ and C_max_ between the 50 and 100 mg BID doses calculated on day 10 increased in a sub-proportional manner with approximately 1.5-fold increases for the twofold increase in dose*.* Statistical analysis confirmed the slight deviation from dose-proportionality, with geometric least squares means of dose- and body weight-normalized AUC_0–τ_ and C_max_ ratios (100 mg:50 mg) of 0.661 and 0.667, respectively, and with the 90% CIs excluding unity (Table 4b).

## Discussion

Galectin-3 inhibitors have been implicated as potential treatments for cancers, fibrotic diseases and other disorders [5]. Efforts to develop galectin-3 binding ligands have included the use of large biomolecules like monoclonal antibodies, natural galactose-based polymers such as pectins, synthetic multivalent ligands and small ligands [29–31]. Many of these however, have inadequate cellular uptake, low binding affinity and/or limited oral bioavailability [32, 33], and the galectin-3 inhibitory effect of pectins, in particular, is not well supported [3, 34]. The small-molecule galectin-3 inhibitor, GB0139, has a high binding affinity and is a potential treatment for IPF [25]. While the inhaled formulation offers targeted and efficient uptake directly in the lungs, and is optimal for IPF treatment, the high polarity of GB0139 results in poor intestinal absorption and prompt elimination if administered systemically [27]. The promising results of GB0139 in fibrotic disease treatment, however, have confirmed galectin-3 as a novel and efficient therapeutic target in humans [25].

GB1211 is an α-D-galactopyranoside with high galectin-3 high binding affinity (K_d_ of 0.025 ± 0.0017 µM) [27] and to our knowledge, is the first orally bioavailable small molecule galectin-3 inhibitor to be tested in humans. Developed to be systemically active, GB1211 may have potential to treat multiple disease types from fibrosis to cancer. The current ascending single- and multiple-oral-dose study, with food-effect, determined the safety, tolerability and PK profile of GB1211 in healthy participants. Overall, 78 individuals participated, 42 of whom were exposed to single oral doses of 5–400 mg GB1211, and 16 who were exposed to BID oral doses of 50–100 mg GB1211 from days 1 to 9, followed by a single dose on day 10. GB1211 was well tolerated, with no severe TEAEs or serious AEs reported. There was no treatment- or dose-related trend and no clinically significant finding in the clinical laboratory evaluations, vital signs data, ECG data or physical examination finding during the study.

Following single and multiple oral doses under fasted conditions, GB1211 was absorbed with median T_max_ ranging from 1.75 to 4 h post-dose. Plasma concentrations of GB1211 decreased in an apparent biphasic manner with an arithmetic mean t_½_ of approximately 11–16 h. Following multiple BID oral dosing, there was approximately a twofold accumulation of GB1211 in plasma, with steady-state being attained within 3 days.

Urinary excretion of unchanged drug represented up to 30% of the administered dose and dosing after a high-fat meal delayed absorption by 2 h but did not markedly affect systemic exposure of GB1211.

Systemic exposure to GB1211 increased in a dose-proportional manner across the 5–50 mg (5 mg capsule) dose range, and in a sub-proportional manner across the 50–400 mg (50 mg capsule) dose range. There was a clear difference in exposure between the 5 and 50 mg capsule formulations with a lower systemic exposure and a slightly delayed T_max_ observed with the 50 mg capsule vs 10 × 5 mg capsules. This was likely a result of slower release from the 50 mg formulation than from the 5 mg formulation as observed in in vitro dissolution assays (unpublished data on file) rather than an intrinsic property of the molecule.

In a recent preclinical study where orally available galectin-3 inhibitors were developed and optimized for the treatment of fibrotic disease, GB1211 was identified as a highly selective galectin-3 inhibitor, with a favorable safety profile against a panel of different targets including enzymes, receptors, and ion channels [27]. In the same study, GB1211 exhibited promise as a therapeutic agent in fibrotic disease by inhibiting TGFβ-induced fibrosis gene expression in vitro in a human liver stellate cell line and reducing fibrosis in vivo in murine models of liver (CCl_4_-induced) and lung (bleomycin-induced) fibrosis. GB1211 also potently inhibited galectin-3 expression on human macrophages in vitro indicating a potential for wider use. In further preclinical studies, a structurally similar galectin-3 inhibitor, GB1107, inhibited lung adenocarcinoma growth and metastasis, and augmented the tumor response to programmed death-ligand 1 blockade [7]. GB1107 has also been shown to inhibit tumor growth in orthotopic gastric cancer-bearing mice by significantly reducing STAT3 and β-catenin activation [35]. Therefore, the high selectivity of GB1211 and therapeutic effect observed by Zetterberg et al. [27], along with the safety and PK findings reported here, suggest GB1211 may be an advantageous treatment option for fibrotic disease, cancer and other galectin-3-mediated conditions.

Inhibition of galectin-3 with GB1211 was not related to any treatment- or dose-related trends and there were no clinically significant safety or tolerability findings. GB1211 displayed favorable PK properties, with low to moderate variability of systemic exposure and minor food-effect on drug disposition. GB1211 is the first orally bioavailable small molecule galectin-3 inhibitor and is a potential novel treatment for diseases associated with high galectin-3 levels. These findings support further clinical development of GB1211.

## Supplementary Information

Below is the link to the electronic supplementary material.

Supplementary file1 (DOCX 100 KB)

## Data Availability

Research data are not shared.
