# Supplementary material for: Safety and pharmacokinetics of GB1211, an oral galectin-3 inhibitor: a single- and multiple-dose first-in-human study in healthy participants
Source: Cancer Chemother Pharmacol. 2023 Mar 13;91(3):267–80. doi: 10.1007/s00280-023-04513-y (PMC10010643; doi:10.1007/s00280-023-04513-y)
Supplement: Supplementary file 1 — Supplementary file1 (DOCX 100 KB) [file 280_2023_4513_MOESM1_ESM.docx]

**Safety and pharmacokinetics of GB1211, an oral galectin-3 inhibitor: a single- and multiple-dose first-in-human study in healthy participants**

**Authors:** Vassilios Aslanis^1*^, Robert J. Slack^1*^, Alison C. MacKinnon^1^, Catherine McClinton^1^, Susan Tantawi^1^, Lise Gravelle^1^, Ulf J. Nilsson^2^, Hakon Leffler^3^, Ashley Brooks^4^, Sanjeev K. Khindri^1^, Richard P. Marshall^1^, Anders Pedersen^1^, Hans Schambye^1^, Fredrik Zetterberg^1^

*Co-leading authors

^1^Galecto Biotech AB, 2200, Copenhagen N, Denmark
^2^_­_Department of Chemistry, Lund University, 22100, Lund, Sweden
^3^Department of Laboratory Medicine, Lund University, 22100, Lund, Sweden
^4^Covance Clinical Research Unit Ltd., Leeds, UK

**Correspondence:**
Robert J. Slack; Galecto Biotech AB, 2200, Copenhagen N, Denmark,
Phone: +44 (0) 7341 079380
Email: [RJSlack@galecto.com](mailto:RJSlack@galecto.com)

**Supplementary Appendix
Full exclusion criteria**

Volunteers were excluded from the study if they met any of the following criteria at the Screening visit unless otherwise stated:

1. Significant history or clinical manifestation of any metabolic, allergic, dermatologic, hepatic, renal, hematologic, pulmonary, cardiovascular, gastrointestinal, neurologic, respiratory, endocrine or psychiatric disorder, as determined by the Investigator (or designee)
2. History of febrile illness within 7 days prior to the first dose of study drug or subjects with evidence of active infection
3. History of significant hypersensitivity, intolerance, or allergy to any drug compound, food, or other substance, unless approved by the Investigator (or designee)
4. History of stomach or intestinal surgery or resection that would potentially alter absorption and/or excretion of orally administered drugs (uncomplicated appendectomy and hernia repair will be allowed, but not cholecystectomy)
5. Any of the following:

Part A

- 1. QTcF >450 msec confirmed by repeat measurement
  2. QRS duration >110 msec confirmed by repeat measurement
  3. PR interval >220 msec confirmed by repeat measurement
  4. findings that would make QTc measurements difficult or QTc data uninterpretable
  5. history of additional risk factors for torsades de pointes (e.g. heart failure, hypokalemia, family history of long QT syndrome)

Part B

1. clinically significant electrocardiogram abnormalities or QTcF >450 msec for males and 470 msec for females at either Screening or Day 1 pre-dose, or any prior history of QT abnormality
2. History of alcoholism or drug/chemical abuse within 1 year prior to Check-in
3. Alcohol consumption of >21 units per week for males and >14 units per week for females. One unit of alcohol equals ½ pint (285 mL) of beer or lager, 1 glass (125 mL) of wine, or 1/6 gill (25 mL) of spirits
4. Positive alcohol breath test result or positive urine drug screen (confirmed by repeat) at Screening or Check-in
5. Positive hepatitis panel and/or positive human immunodeficiency virus test
6. Participation in a clinical study involving administration of an investigational agent or vaccine (new chemical entity) or having received a biological product in the past 90 days prior to dosing
7. Use or intend to use any medications/products known to alter drug absorption, metabolism, or elimination processes, including St. John’s wort, within 30 days prior to dosing, unless deemed acceptable by the Investigator (or designee)
8. Use or intend to use any prescription medications/products other than hormone replacement therapy, oral, implantable, transdermal, injectable, or intrauterine contraceptives within 14 days prior to dosing, unless deemed acceptable by the Investigator (or designee)
9. Use or intend to use slow-release medications/products considered to still be active within 14 days prior to Check-in, unless deemed acceptable by the Investigator (or designee)
10. Use or intend to use any non-prescription medications/products including vitamins, minerals, and phytotherapeutic/herbal/plant-derived preparations within 7 days prior to Check-in, unless deemed acceptable by the Investigator (or designee)
11. Use of tobacco- or nicotine-containing products within 3 months prior to Check-in, or positive cotinine at Screening or Check-in
12. Receipt of blood products within 2 months prior to Check-in and donation of blood from 3 months prior to Screening, plasma from 2 weeks prior to Screening, or platelets from 6 weeks prior to Screening
13. Poor peripheral venous access
14. Have previously completed or withdrawn from this study investigating GB1211, and have previously received the investigational product
15. Subject who, in the opinion of the Investigator (or designee), should not participate in this study

**Determination of the food-effect**

To determine the food-effect, a two-period design was chosen, so that a within-participant assessment of the influence of food on the pharmacokinetics of GB1211 could be performed. The least square (LS) means were calculated for the fed and fasted treatments, and mean differences were calculated between them. The residual variance from the mixed model was used to calculate 90% and 95% confidence intervals (CIs) for the difference between the fed and fasted treatments. These values were back-transformed to give geometric (LS) means, a point estimate, and 90% and 95% CIs for the ratio of the fed treatment relative to the fasted treatment. Within-participant coefficients of variation (CVW) were calculated based on the log-normal distribution using the following formula: CVW(%) = [exp(mse) – 1]½ x 100, where mse is the residual error from the mixed model.

**Supplementary Tables**

**Table S1** Treatment-emergent adverse events by System Organ Class for Part A (a) and Part B (b) – safety population

(a)

| MedDRA system organ class preferred term, *n* (%)  [no. of AEs] | | Placebo | |  | GB1211 | | | | | | | |
| --- | --- | --- | --- | --- | --- | --- | --- | --- | --- | --- | --- | --- |
|  |  | Fasted (*n* = 14) | Fed  (*n* = 2) |  | 5 mg,  fasted (*n* = 6) | 20 mg, fasted (*n* = 6) | 50 mg  (10 x 5 mg), fasted (*n* = 6) | 50 mg (1 x 50 mg), fasted  (*n* = 6) | 50 mg (1 x 50 mg), fed  (*n* = 6) | 100 mg, fasted (*n* = 6) | 200 mg, fasted (*n* = 6) | 400 mg, fasted (*n* = 6) |
| Overall total | | 5 (35.7%) [5] | 1 (50.0%) [1] |  | 3 (50.0%) [4] | 1 (16.7%) [1] | 1 (16.7%) [1] | 1 (16.7%) [1] | 1 (16.7%) [1] | 1 (16.7%) [2] | 0 | 1 (16.7%) [1] |
| Gastrointestinal disorders | | 2 (14.3%) [2] | 0 |  | 1 (16.7%) [1] | 0 | 0 | 0 | 0 | 1 (16.7%) [1] | 0 | 0 |
| Constipation | | 1 (7.1%) [1] | 0 |  | 0 | 0 | 0 | 0 | 0 | 0 | 0 | 0 |
| Diarrhea | | 0 | 0 |  | 1 (16.7%) [1] | 0 | 0 | 0 | 0 | 0 | 0 | 0 |
| Dry mouth | | 1 (7.1%) [1] | 0 |  | 0 | 0 | 0 | 0 | 0 | 0 | 0 | 0 |
| Dyspepsia | | 0 | 0 |  | 0 | 0 | 0 | 0 | 0 | 1 (16.7%) [1] | 0 | 0 |
| Skin and subcutaneous tissue disorders | | 0 | 1 (50.0%) [1] |  | 0 | 0 | 0 | 0 | 1 (16.7%) [1] | 0 | 0 | 1 (16.7%) [1] |
| Dry skin | | 0 | 1 (50.0%) [1] |  | 0 | 0 | 0 | 0 | 1 (16.7%) [1] | 0 | 0 | 0 |
| Rash | | 0 | 0 |  | 0 | 0 | 0 | 0 | 0 | 0 | 0 | 1 (16.7%) [1] |
| General disorders and administration site conditions | | 0 | 0 |  | 1 (16.7%) [1] | 1 (16.7%) [1] | 0 | 0 | 0 | 0 | 0 | 0 |
| Medical device site reaction | | 0 | 0 |  | 1 (16.7%) [1] | 0 | 0 | 0 | 0 | 0 | 0 | 0 |
| Pain | | 0 | 0 |  | 0 | 1 (16.7%) [1] | 0 | 0 | 0 | 0 | 0 | 0 |
| Infections and infestations | | 0 | 0 |  | 1 (16.7%) [1] | 0 | 0 | 0 | 0 | 1 (16.7%) [1] | 0 | 0 |
| Nasopharyngitis | | 0 | 0 |  | 1 (16.7%) [1] | 0 | 0 | 0 | 0 | 1 (16.7%) [1] | 0 | 0 |
| Musculoskeletal and connective tissue disorders | 0 | | 0 |  | 1 (16.7%) [1] | 0 | 1 (16.7%) [1] | 0 | 0 | 0 | 0 | 0 |
| Back pain | | 0 | 0 |  | 1 (16.7%) [1] | 0 | 1 (16.7%) [1] | 0 | 0 | 0 | 0 | 0 |
| Injury, poisoning and procedural complications | | 1 (7.1%) [1] | 0 |  | 0 | 0 | 0 | 0 | 0 | 0 | 0 | 0 |
| Wound | | 1 (7.1%) [1] | 0 |  | 0 | 0 | 0 | 0 | 0 | 0 | 0 | 0 |
| Metabolism and nutrition disorders | | 1 (7.1%) [1] | 0 |  | 0 | 0 | 0 | 0 | 0 | 0 | 0 | 0 |
| Decreased appetite | | 1 (7.1%) [1] | 0 |  | 0 | 0 | 0 | 0 | 0 | 0 | 0 | 0 |
| Nervous system disorders | | 1 (7.1%) [1] | 0 |  | 0 | 0 | 0 | 0 | 0 | 0 | 0 | 0 |
| Headache | | 1 (7.1%) [1] | 0 |  | 0 | 0 | 0 | 0 | 0 | 0 | 0 | 0 |
| Respiratory, thoracic and mediastinal disorders | | 0 | 0 |  | 0 | 0 | 0 | 1 (16.7%) [1] | 0 | 0 | 0 | 0 |
| Cough | | 0 | 0 |  | 0 | 0 | 0 | 1 (16.7%) [1] | 0 | 0 | 0 | 0 |

(b)

| MedDRA system organ class preferred term, *n* (%) [no. of AEs] | Placebo BID  (*n* = 6) | GB1211 | |
| --- | --- | --- | --- |
|  |  | 50 mg BID (*n* = 8) | 100 mg BID (*n* = 8) |
| Overall total | 1 (16.7%) [2] | 4 (50.0%) [8] | 3 (37.5%) [8] |
| Nervous system disorders | 0 | 4 (50.0%) [5] | 0 |
| Headache | 0 | 4 (50.0%) [5] | 0 |
| Gastrointestinal disorders | 1 (16.7%) [2] | 0 | 2 (25.0%) [4] |
| Constipation | 1 (16.7%) [1] | 0 | 2 (25.0%) [2] |
| Abdominal distension | 0 | 0 | 1 (12.5%) [1] |
| Abdominal pain | 1 (16.7%) [1] | 0 | 0 |
| Abdominal pain upper | 0 | 0 | 1 (12.5%) [1] |
| Renal and urinary disorders | 0 | 1 (12.5%) [1] | 2 (25.0%) [2] |
| Pollakiuria | 0 | 0 | 2 (25.0%) [2] |
| Dysuria | 0 | 1 (12.5%) [1] | 0 |
| Musculoskeletal and connective tissue disorders | 0 | 0 | 1 (12.5%) [2] |
| Arthralgia | 0 | 0 | 1 (12.5%) [1] |
| Pain in extremity | 0 | 0 | 1 (12.5%) [1] |
| Injury, poisoning and procedural complications | 0 | 1 (12.5%) [1] | 0 |
| Limb injury | 0 | 1 (12.5%) [1] | 0 |
| Reproductive system and breast disorders | 0 | 1 (12.5%) [1] | 0 |
| Menstruation irregular | 0 | 1 (12.5%) [1] | 0 |

*AE* adverse event; *BID* twice daily; *MedDRA* Medical Dictionary for Regulatory Activities; *n* number of participants
Events were coded using MedDRA Version 21.1

**Table S2** Statistical assessment of the effect of food on pharmacokinetic parameters of GB1211 – Part A

| Parameter | Treatment | N | Geometric LS means | Ratio of geometric  LS means  (Fed : Fasted) | 90% CI for  the ratio  (Fed : Fasted) | | 95% CI for  the ratio  (Fed : Fasted) | | Within-participant CV% |
| --- | --- | --- | --- | --- | --- | --- | --- | --- | --- |
|  |  |  |  |  | Lower | Upper | Lower | Upper |  |
| AUC_0–∞_ (h*ng/mL) | Fasted Fed | 6  6 | 4520  5420 | 1.20 | 0.940 | 1.53 | 0.878 | 1.64 | 21.3 |
| AUC_0–tlast_ (h*ng/mL) | Fasted  Fed | 6  6 | 4180  4930 | 1.18 | 0.909 | 1.53 | 0.845 | 1.65 | 22.8 |
| C_max_ (ng/mL) | Fasted  Fed | 6  6 | 358  389 | 1.09 | 0.789 | 1.50 | 0.722 | 1.63 | 28.0 |

*AUC_0–tlast_* area under the plasma concentration-time curve from time 0 to the time of last quantifiable concentration; *AUC_0–∞_* area under the plasma concentration-time curve from time zero to infinity; *C_max_* maximum observed plasma concentration; *CI* confidence interval; *CV* coefficient of variation; *LS* least-squares

**Supplementary Figures
Fig. S1** Study design.

| **a**  **Cohorts (not including the food-effect cohort)** 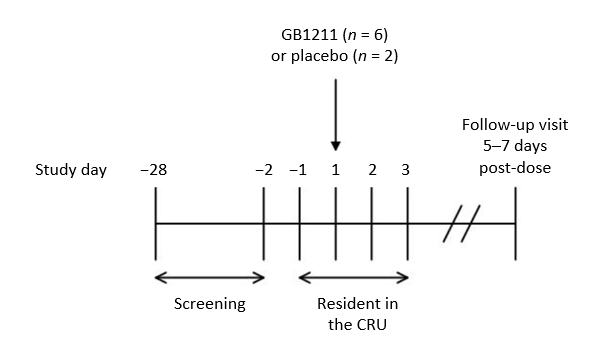  **Food-effect cohort** 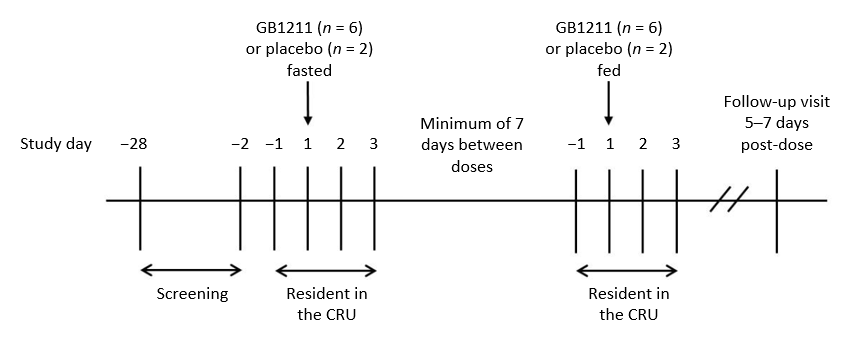 |
| --- |
| **b**  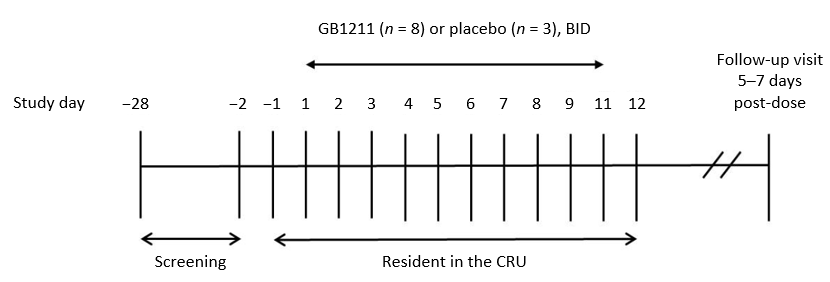 |

*BID* twice daily; *CRU* Clinical Research Unit; *n* number of participants
